# Better to light a candle than curse the darkness: illuminating spatial localization and temporal dynamics of rapid microbial growth in the rhizosphere

**DOI:** 10.3389/fpls.2013.00323

**Published:** 2013-09-02

**Authors:** Patrick M. Herron, Daniel J. Gage, Catalina Arango Pinedo, Zane K. Haider, Zoe G. Cardon

**Affiliations:** ^1^Department of Ecology and Evolutionary Biology, University of ConnecticutStorrs, CT, USA; ^2^Department of Molecular and Cell Biology, University of ConnecticutStorrs, CT, USA; ^3^Marine Biological Laboratory, The Ecosystems CenterWoods Hole, MA, USA

**Keywords:** rhizosphere, microbiosensor, *lux*, roots, *Pseudomonas*, *Zea mays*, *Solanum lycopersicum*, *Populus nigra*

## Abstract

The rhizosphere is a hotbed of microbial activity in ecosystems, fueled by carbon compounds from plant roots. Basic questions about the location and dynamics of plant-spurred microbial growth in the rhizosphere are difficult to answer with standard, destructive soil assays mixing a multitude of microbe-scale microenvironments in a single, often sieved, sample. Soil microbial biosensors designed with the *luxCDABE* reporter genes fused to a promoter of interest enable continuous imaging of the microbial perception of (and response to) environmental conditions in soil. We used the common soil bacterium *Pseudomonas putida* KT2440 as host to plasmid pZKH2 containing a fusion between the strong constitutive promoter *nptII* and *luxCDABE* (coding for light-emitting proteins) from *Vibrio fischeri*. Experiments in liquid media demonstrated that high light production by KT2440/pZKH2 was associated with rapid microbial growth supported by high carbon availability. We applied the biosensors in microcosms filled with non-sterile soil in which corn (*Zea mays* L.), black poplar (*Populus nigra* L.), or tomato (*Solanum lycopersicum* L.) was growing. We detected minimal light production from microbiosensors in the bulk soil, but biosensors reported continuously from around roots for as long as six days. For corn, peaks of luminescence were detected 1–4 and 20–35 mm along the root axis behind growing root tips, with the location of maximum light production moving farther back from the tip as root growth rate increased. For poplar, luminescence around mature roots increased and decreased on a coordinated diel rhythm, but was not bright near root tips. For tomato, luminescence was dynamic, but did not exhibit a diel rhythm, appearing in acropetal waves along roots. KT2440/pZKH2 revealed that root tips are not always the only, or even the dominant, hotspots for rhizosphere microbial growth, and carbon availability is highly variable in space and time around roots.

## Introduction

Most terrestrial plants grow in environments where restricted quantities of water or mineral nutrients (e.g., nitrogen, phosphorous) limit plant growth. Plants invest a significant amount of fixed carbon into root tissue and rhizodeposition to acquire these limiting resources. As roots grow, they release carbon, in the process stimulating the growth and activities of surrounding microbial community (Wardle, 1992; Cheng et al., 1996). van Veen et al. (1991) estimate, for example, that for every 10 grams of carbon assimilated by a plant, an estimated 4 grams are contributed to the soil as rhizodeposition. These rhizodeposits provide energy supporting growth and activity of microbes in the rhizosphere (Lynch and Whipps, 1990). Microbial growth and activity, in turn, affect nutrient availability to plants, via immobilization of nutrients into microbial biomass, release of mineral nitrogen during decomposition of organic matter, or via a soil “microbial loop” in which protozoa grazing on rhizosphere microbes release waste ammonium (Helal and Sauerbeck, 1983; Clarholm, 1985; Bottner et al., 1988; Dormaar, 1990; Wheatley et al., 1990; Darrah, 1991; Haider et al., 1991; De Nobili et al., 2001; Kuzyakov, 2002; Cardon and Gage, 2006; Jones et al., 2009; Kuzyakov and Xu, 2013).

Basic questions about plant-spurred microbial growth and activity in the rhizosphere are difficult to answer with standard, destructive soil assays that mix a multitude of microbe-scale microenvironments in a single, often sieved, sample. Is the energy contribution from roots to microbes a one-time occurrence as the root tip passes by in the soil, or do plants continue to release carbon at the same location again and again? Do known shoot-root carbon allocation patterns in various plant species translate to similar temporal (or spatial) patterns of carbon availability to free-living rhizosphere microbes? Living soil microbial biosensors, engineered to “report” conditions in their local microenvironment (and/or their response to those conditions), offer the possibility of gathering the continuous *in situ* information necessary to begin answering such questions.

Microbial biosensors consist of a host strain (usually bacterial) that contains inserted DNA (on the chromosome or a plasmid), coding for an environmentally controlled promoter, driving expression of an easily assayed reporter gene (e.g., *inaZ*, *gfp*, *lux*) (Hansen and Sørensen, 2001; Gage et al., 2008). The expression of the reporter molecule is thus tied to the activity of the promoter within the host organism. The ability to choose a promoter that scales with a metabolic process or is activated by a specific compound in the environment contributes to the great flexibility of biosensors. Investigators have made use of this flexibility to investigate pollutants such as naphthalene (King et al., 1990) and PCB's (Boldt et al., 2004), water potential around plant roots (Herron et al., 2010), as well as more common soil and root-derived compounds such as sucrose and tryptophan (Jaeger et al., 1999), nitrate (DeAngelis et al., 2005), and galactoside sugars (Bringhurst et al., 2001). Common reporter systems include a number that require destructive harvest for measure (*lacZ*, *phoA*, and *inaZ*) as well as a number that yield a visible readout (e.g., *gfp*, *lux*). The value of microbial biosensors as measurement devices is tied to the great numbers that can be applied to a system, design flexibility, the sensitivity of microbes to low activities of inducing signal (Hansen and Sørensen, 2001) and the specificity of the biosensor to “bioavailable” forms of that signal.

We used the common soil bacterium *Pseudomonas putida* KT2440 to host plasmid pZKH2, which contains a fusion between the neomycin phosphotransferase II (*nptII*) promoter, cloned from Tn5 (Rothstein et al., 1980; Axtell and Beattie, 2002; Herron et al., 2010), and *lux* reporter genes cloned from the marine bacterium *Vibrio fisheri*. Bioluminescence, while quite common in marine bacterial species, is very rare in terrestrial organisms (Stewart and Williams, 1992). The application of marine *lux* genes under control of known promoters and incorporated into terrestrial bacteria offers the opportunity to track luminescence from these specific bacteria in a dark soil system. The *nptII* promoter in plasmid pZKH2 is constitutive, in the transposon Tn5 it drives the expression of genes that confer antibiotic resistance, and *nptII* has been shown to function in a large number of bacterial species (Labes et al., 1990; Joyner and Lindow, 2000; Wright and Beattie, 2004) isolated from multiple environments. In *Pseudomonas* species, P*nptII* has been reported to drive transcription at moderate levels under a variety of conditions (Axtell and Beattie, 2002; Wright and Beattie, 2004; Goymer et al., 2006; Herron et al., 2010; Park et al., 2010).

Initial results showed *P. putida* KT2440 carrying the pZKH2 plasmid (KT2440/pZKH2) produces light only when growing rapidly. Luminescence does not indicate the presence/absence of particular compounds; instead, luminescence signals the integrated microbial perception of the local environment, indicating sufficient energy and substrates are available to support microbial growth and light production as well.

We characterized the behavior of the light emission response from the biosensors using traditional growth curve studies, pulsed carbon availability experiments, and substrate amendments into soil. The biosensor was then applied into plant-soil microcosms planted with species known from the literature to have distinct internal vascular architectures and/or root and carbon allocation patterns. Corn (*Zea mays* L.) is well-known to elongate rapidly in soil and produce abundant mucilage and other rhizodeposits at the growing root tip (see McCully, 1999). We explored spatial and temporal patterns in KT2240/pZKH2 luminescence near corn root tips growing at different rates. Members of the genus *Populus* are known to store newly-fixed carbon in starch during the day, then break it down end of day for shipment to roots, producing a strong diel oscillation in belowground carbon allocation (Dickson, 1991). We explored whether *Populus nigra* L. stimulated rhythmic luminescence from KT2240/pZKH2 over several diel cycles. Finally, tomato (*Solanum lycopersicum* L.) is known to have highly modular internal vascular architecture (e.g., Zanne et al., 2006), with root-shoot physiological units operating relatively independently. We explored whether biosensor luminescence exhibited coordinated temporal or spatial patterns among roots, or through time. Rather than using a biosensor to detect the presence of a pre-determined substrate (e.g., galactosides, Bringhurst et al., 2001) or condition (e.g., water potential, Herron et al., 2010), the KT2240/pZKH2 biosensor design has the advantage of reporting periods of rapid growth on any substrate the microbe can use (exudates, secretions, rhizodeposited cap cells), without requiring identification or quantification of particular substrate components.

## Methods

### Plasmid construction

*E coli* strains XL1Blue MRF'(Stratagene) and XL1Blue MRF'Km were used as hosts for all plasmids. The strains were grown in LB broth or plates with antibiotics as needed (tetracycline 10 μg ml^−1^; amplicillin 100 μg ml^−1^; kanamycin 25 μg ml^−1^). All electroporations were at 1.8 kV. Plasmids were isolated from LB cultures using a Qiagen Miniprep kit (Qiagen).

Joerg Graf (University of Connecticut) generously provided the plasmid pLM2819 (Stewart and McCarter, 2003) containing the full *luxCDABE* cassette cloned from *Vibrio fisheri*. Initially, the *lux* operon was cut from pLM2819 with KpnI and cloned into a KpnI-cut pBluescriptSK(−), resulting in pDG115. A *trp* terminator was cloned into the XbaI site of pDG115, upstream of the *lux* operon, to make pDG117.

**Figure 1:**
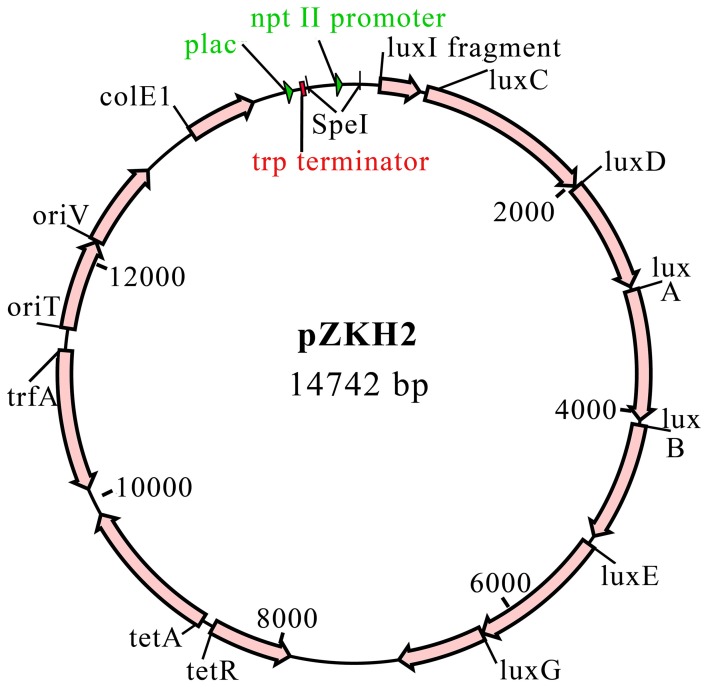
**Plasmid pZKH2 with *nptII-luxCDABE* fusion**.

A promoterless-*lux* construct, pCAP40, was created by excising the *trp* terminator-*lux* fragment from pDG117 as a KpnI-XbaI fragment and ligating it into plasmid pCM62 (Marx and Lidstrom, 2001). The *nptII* promoter for the *nptII-lux* construct was amplified from Tn5 by PCR using the primers 5′ GGACTAGTGTCAGGCTGTTACAGCTC 3′ and 5′ CTACTAGTTCATGCGAAACGATCCTC 3′. These primers include SpeI restriction sites (underlined). The *nptII* fragment was cloned into pGEM T-Easy (Promega). This plasmid was digested with SpeI and the *nptII* fragment was ligated to SpeI-cut, dephosphorylated pCAP40. The resulting plasmid was called pZKH2 (Figure 1).

### Bacterial strains, growth media and chemicals

Both plasmids pZKH2 and pCAP40 were moved by triparental mating into *P. putida* KT2440 using plasmid pRK600 as the helper plasmid. In preparation for broth and soil-based experiments, *P. putida* KT2440/pZKH2 and *P. putida* KT2440/pCAP40 were streaked on LB Tetracycline (10 μg ml^−1^) plates and grown at 30°C.

For broth experiments, bacteria were grown in M9 minimal medium (Sambrook et al., 1989) amended with citrate, succinate, or glucose (0.15–0.4% w/v). All cultures were amended with tetracycline at 10 μg ml^−1^ unless noted otherwise. Bacterial strains were inoculated into 2.5 ml of medium in 18 × 150-mm tubes and grown at 30°C with constant shaking (120 rpm). In cases when bacteria were needed for testing in peristaltic experiments and soil chambers, a single colony was inoculated into 20 ml of M9 medium with tetracycline and glucose (0.15–0.4% w/v) and grown in 250 ml Ehrlenmyer flasks at 30°C with constant shaking (120 rpm). Optical density values are reported for cultures measured in 96-well or 48-well plates. For comparison with optical densities measured in a standard cuvette with a 1 cm pathlength, optical densities from these well plates should be scaled with a multiplier dependent on path length. For example, the path length of the medium is approximately 1/3 of a cm for 100 μl inoculations in 96-well plates. Optical densities should be multiplied by 3 in order to compare 96-well plate data to cultures measured in a standard cuvette with a 1 cm pathlength.

### Plate reader experiments

#### Influence of growth phase on light production

KT2440/pZKH2 and KT2440/pCAP40 were grown as described above until mid-exponential phase in M9 medium, then centrifuged, washed in M9 medium three times, and re-suspended to an optical density of 0.1 OD_595_. Bacteria were inoculated in triplicate, to 0.005 OD_595_, into the wells of a Falcon 48-well plate (BD Biosciences, FranklinLakes, NJ) filled with 200 μL of M9 amended with tetracycline. For KT2440/pZKH2, triplicate wells were amended with citrate (0.1, 0.2, 0.4, 0.6% w/v) or with succinate (0.4%, 0.6%). For KT2440/pCAP40, only citrate (0.1, 0.2, 0.4, 0.6% w/v) was tested. Bacteria were grown at 30°C and shaken every 5 min in a plate reader (Synergy HT Multi-Detection Microplate Reader, Biotek). Optical density was measured every 5 min at 595 nm and luminescence was read every 30 min from the bottom of the plate with sensitivity set at 125, until stationary phase was reached. The software packages used to alternate optical density and luminescence measurements were Automate 4 (Network Automation, Los Angeles) in conjunction with KC4 (Biotek, Winooski, VT). Specific luminescence was calculated as the light emission measured by the Synergy plate reader in each well divided by the optical density of the suspension of bacteria in that well.

#### Influence of carbon, M9, and oxygen availability on light production

In 48-well plate experiments, a sharp decline in light production was consistently observed in late exponential growth. Normalized light production dropped to zero in stationary phase. We tested whether oxygen, mineral nutrient, or carbon (energy) limitations were the cause of the decline.

Six independent cultures of KT2440/pZKH2 were grown as inoculant, three in M9 amended with citrate (0.15% w/v), and three in M9 amended with glucose (0.15% w/v), all with tetracycline (10 μg ml^−1^). At mid-exponential growth, cells were spun down, washed in M9 medium three times, then each independent culture was re-suspended to an optical density of 0.1 in M9 minimal medium (no carbon). Ten microliters (10 μl) of bacterial suspensions were inoculated into 190 μl of M9 medium amended with glucose (0.1% w/v) or citrate (0.1% w/v), in a 48 well plate.

Three hundred and eighty minutes after dilution into fresh medium, M9+glucose cultures were in late exponential growth (and specific luminescence had just begun to decline); M9+citrate cultures were in stationary phase and luminescence had declined to zero. The 48-well plate was removed from the plate reader. Triplicate wells were assigned to oxygen, mineral nutrient, carbon and control treatments. A pipette was used to bubble air in the wells that received an oxygen amendment. Carbon amendment wells received a boost of 0.3% carbon source of the same type they had been growing in (3 μl addition of 20% w/v citrate or glucose). Mineral nutrient wells received a 50% increase in M9 nutrients (10 μl addition of 10× M9 salts). Control wells received 3 μl of distilled and deionized water. The plate was returned to the plate reader and OD595 and luminescence tracked for another 600 min.

### Experiments on filter discs

#### Dynamic response to pulsed carbon availability

KT2440/pZKH2 was grown in 20 ml of M9 amended with tetracycline (10 μg ml^−1^) and glucose (0.15% w/v). This low concentration of glucose was used to minimize the amount of energy stored within the bacteria as polyhydroxyalkanoates (PHAs) prior to the experiment (Huijberts et al., 1992). After reaching stationary phase (~0.08 OD) the bacteria were spun down, washed three times and resuspended in M9 to a concentration of 0.005 OD in a flask with 20 ml of M9 minimal medium without tetracycline or carbon. The culture was incubated for another 24 h to help exhaust energy supplies. Bacteria were re-suspended to an optical density of 0.0001 for the experiment.

2.5 ml of the bacterial suspension was filtered onto each of five disposable 26-mm 0.45-micron syringe filters (Corning Cat. 431220, Fisher Scientific). One control filter was not inoculated with bacteria to serve as a blank. All filters were flushed with 30 ml of M9 (no carbon) to remove any contaminants that could serve as a carbon source. All filters were arranged on a board with individual pieces of Tygon tubing fitted to the front and rear of the filters. The board with filters was placed inside a light tight box at a distance of 15 cm in front of a Princeton Instruments Versarray CCD camera. This camera contained a 1024 × 1024 back-thinned chip cooled to −70°C (Acton PI 1 kb Versarray Cooled Camera, Princeton Instruments, Trenton, NJ) fitted with a 25 mm (0.95 na) lens (Universe Kogaku, Oyster Bay, NY). The tubes leading into each of the filters connected to lines on a multi-channel peristaltic pump (Zellweger Analytics, Suffolk, GB) that delivered a steady flow of M9 solution at a rate of 0.5 ml min^−1^ (no carbon added, no tetracycline added). Filters + bacteria equilibrated for 12 h.

The filters that had received bacteria were broken into two treatments: Treatment 1 (2 replicates), bacteria received a constant flow of M9 for the duration of the experiment; Treatment 2 (3 replicates), bacteria received a flow of M9, followed by a pulse of M9 amended with glucose (0.1%) for 180 min, followed by a return to M9.

Images were captured with the Versarray CCD camera every 5 min for the duration of the experiment and pixels were binned 10 × 10. A background image was obtained prior to the experiment and subtracted from each image. The camera was controlled using WinView/32 software (Princeton Instruments). Total light emitted for each filter disc was analyzed for each image using a macro written for ImageJ v 1.38 (Rasband, 1997--2007).

### Experiments in soil – no plants

Soil used in both soil experiments without plants was a 1:1 mix of sand and Connecticut loam obtained from the Ecology and Evolutionary Biology Greenhouses at the University of Connecticut, Storrs and sieved to 2 mm.

#### Detection of biosensor luminescence in soil supported by distinct carbon substrates

We tested whether we could detect luminescence from the biosensor inoculated into soil with three different labile, low molecular weight carbon substrates. KT2440/pZKH2 was grown over 48 h in M9 and tetracycline (10 μg ml^−1^) amended with 0.4% citrate, 0.4% glucose, or 0.4% acetate (w/v) at 30°C. The cultures were then spun down, washed three times with M9, and re-suspended to three optical densities (0.02, 0.04, 0.08) in each of the three media treatments. The citrate-amended M9 culture was also used as the source of bacteria for a fourth treatment—M9 minimal medium with no added carbon. Twenty-four Eppendorf tubes were each filled with 200 ± 10 mg of soil. For each of two replicates of each M9+carbon combination and optical density, 40 μl of bacterial suspension was added to the soil in the Eppendorf tube. The soil was then mixed with a dissecting needle and vortexed to achieve an even mixture of cell suspension and soil. Soils were then deposited into the wells of a 96-well plate. A glass cover slip, treated with Sea-Drops Anti Fog solution (McNett Corp., Bellingham, WA) was placed over the wells. The plate was placed in a dark box 15 cm in front of a CCD camera (Retiga EX CCD camera, 12 bit 1360 × 1360 pixels, QImaging, Burnaby, BC) with a Navitar TV Zoom 7000 lens. Sixteen minute exposures were captured at 3 × 3 binning every 40 min, for 18 h. The camera was controlled by Openlab software (Improvision) on a Macintosh G4. Images were analyzed using NIH Image v 1.37.

#### Response of biosensor luminescence in soil to repeated pulses of carbon and mineral nutrients

Building on the plate reader experiment (detailed above) testing the influence of carbon and M9 on light production, biosensors were inoculated into soil and luminescence monitored during repeated pulses of added carbon and various mineral nutrients.

Bacteria were grown in M9 minimal medium amended with glucose (0.4% w/v) and tetracycline (10 μg ml^−1^) into stationary phase. Bacteria were spun down and washed two times before being re-suspended in distilled water to an optical density of 0.08. The 1:1 sand and loam mix was packed into a 20 cm (wide) × 27.5 cm (tall) × 2.5 cm (thick) container. The surface of the soil was sprayed with 2 ml of KT2440/pZKH2 suspended in distilled water using a 25 ml reagent sprayer (Kontes, Vineland, NJ USA), then covered with a 20 × 27.5 cm sheet of borosilicate glass for 24 h. Following the 24 h, twenty-two 13-mm nylon filter discs (Millipore, Boston, MA) were attached to the soil surface using stainless steel pins. The discs served as the vehicle for delivering nutrients to soil bacteria underneath them.

Seven treatments (six replicated three times and one treatment, #7, below, replicated four times) were applied in a random pattern among the 22 filter discs. All pre-treatments were pipetted onto the filter discs in 20 μl volumes and left for 48 h; mineral nutrients were added at the same concentrations as found in M9 minimal medium. The seven pre-treatments were:

1. Carbon (as glucose C_6_H_12_O_6_, 0.4% w/v)
2. Nitrogen (as NH_4_Cl)
3. Sulfate (as MgSO_4_)
4. Phosphorous (as a mix of Na_2_HPO_4_ and NaH_2_PO_4_ to yield pH of 6.2)
5. Nitrogen, sulfate, phosphorous (as above)
6. Phosphorous, sulfate, carbon (as above)
7. M9 + Carbon (M9 with glucose 0.4% w/v)

Following the 48 h initial exposure to these pre-treatments, each filter disc received a daily aliquot of nitrogen, phosphorous, carbon or water. We used the PI Versarray camera to capture images of the soil with discs at 115 min exposures with 2 × 2 binning, over 100 h.

### Experiments in soil – soil microcosms with plants

Soil was prepared by using a 1:1:1 mix of loam, sand, and peat obtained from the Ecology and Evolutionary Biology Greenhouses at the University of Connecticut, Storrs. Soil was not sterilized, and was packed into tall, thin microcosms measuring 1 cm (thick, from glass to back) × 22 cm (wide) × 27 cm (tall). Soil in the chamber was brought to approximately 20% soil moisture and lightly fertilized with Peters Professional 20-20-20 fertilizer.

Seeds of four species were used in experiments: *Zea mays* L. (sweet corn) (Kandy Korn “EH,” The Page Seed Company, Greene, NY), *Solanum lycopersicum* L. (tomato) (Celebrity F1, Johnny's Selected Seeds, Winslow, ME), *Capsicum annuum* L. (green pepper) (Yolo, B + T World Seeds, Aigues-Vives, France), and *Artemisia tridentata* var. *vaseyana* (mountain sagebrush, grown from seed gathered in northern Utah, USA). Cuttings of poplar (*Populus nigra* L.) were generously provided by Dr. Rachel Spicer, Harvard University.

Seeds were surface sterilized by soaking in 50% ethanol (1 min) followed by 10% bleach (5 min), then rinsing twice in sterile DI. Seeds germinated on an agar plate until the radical had emerged, then were planted at 1 cm depth in soil. A glass sheet was placed over the soil surface and clamped into place using large binder clips. Tinfoil and cardboard were placed over the surface of the glass sheets prevent light from reaching roots. Microcosms were placed in the greenhouse leaning at a 30° angle from vertical with the glass side face down to promote growth of long roots through soil at the surface of the glass.

Following 10–14 days of growth, microcosms were removed from the greenhouse and the volume of soil was doubled to allow new roots that emerged near the shoot-root interface to grow toward depth in fresh soil. The glass sheet was removed and a short, clear acetate sheet was placed on the surface of the existing soil, extending from the base of the microcosm up to within 5 cm of the top of the soil. One cm thick strips of neoprene foam gasket were positioned to double the thickness of the soil chamber, and an additional 1 cm of soil was added and moistened with a general household sprayer with tap water. The glass and clamps were returned to the microcosm and the microcosm was returned to the greenhouse. Plants were inspected every 2–3 days and when new roots were growing in the chamber, the microcosm was chosen for application of bacteria. The limited number of roots growing in the fresh soil simplified the analysis of patterns of luminescence from bacteria located near individual roots, particularly for work with corn. Tomato and poplar root systems were more complex.

Bacteria were grown in 20 ml of M9 medium amended with glucose (0.4% w/v) and tetracycline (10 μg ml^−1^) in 250 ml flasks. Bacteria were spun down and re-suspended in 15 ml of M9 medium to an optical density of 0.08 OD without tetracycline and without carbon. For application of KT2440/pZKH2, the glass was removed from the side of the chamber and a 25 ml reagent sprayer (Kontes, Vineland, NJ USA) was used to apply an even distribution of bacteria onto the exposed surface of the roots and soil. Following the application of the bacteria, a clear perforated bread bag (25 holes per square cm, Whole Foods, Austin, TX USA) was attached in front of the soil to support the soil and allow air flow.

The microcosm was placed in a 60 × 40 × 40 cm Rubbermaid container (Newell Rubbermaid, Atlanta GA) that had been made light tight using a combination of black duct tape and darkroom cloth material. The soil surface faced the Versarray CCD camera lens at approximately 15 cm distance. Heat produced by the camera required that the camera body itself be placed outside of the box. A 15 × 15 cm piece of sheet metal was machined with a hole that allowed the threading of the C-mount lens to extend through and thread into the body of the camera. The sheet metal was connected to the sides of the light-tight box with two layers of blackroom cloth and black duct tape. This connection allowed the camera and lens position to be moved relative to the position of roots growing down the surface of the soil, but did not allow light into the box. The stem and leaves of the plant extended up out of the box through a hole so that the aboveground portion of the plant was exposed to light from a halogen lamp on a 12 h light/dark cycle. The light was filtered through 5 cm of water to remove infrared heat. A light-tight seal was made with Play-Doh (Hasbro, Pawtucket, RI) kept from drying using parafilm. Reflective insulation (Reflectix, Markleville, IN) on top of the box also kept halogen light from heating up the box. Temperature inside the box was monitored using a temperature logger (Onset Corporation, Bourne, MA USA) and maintained within 1°C between dark and light cycles.

Images of luminescence were captured as 55 min exposures with 1 × 1 or 2 × 2 binning. Following every 55 min exposure, a timer turned on a very weak indiglo nightlight inside the box (AmerTac Model E-22A, generating <1 μmol photons m^−2^ s^−1^ as measured by a LiCor 250A meter equipped with LiCor 190A PAR sensor). While the indiglo light was on, the camera captured a 3-second bright-field image of the microcosm's soil-root surface. This alternating image acquisition was programmed using a macro in the WinView/32 imaging software, for 3–7 days of plant growth. Images were analyzed using ImageJ. For corn, light was quantified in 1 mm or 2 mm increments along the length of individual roots in areas of 25 pixels, up to 90 mm back from root tips, over time. For tomato, a complex root system developed prior to application of bacteria and the whole system was imaged over time. For poplar, individual regions of interest approximately 1 cm in length were selected from mature, non-woody roots at most several days old, and luminescence was quantified through time.

## Results

### Plate reader experiments

#### Influence of growth phase on light production

**Figure 2:**
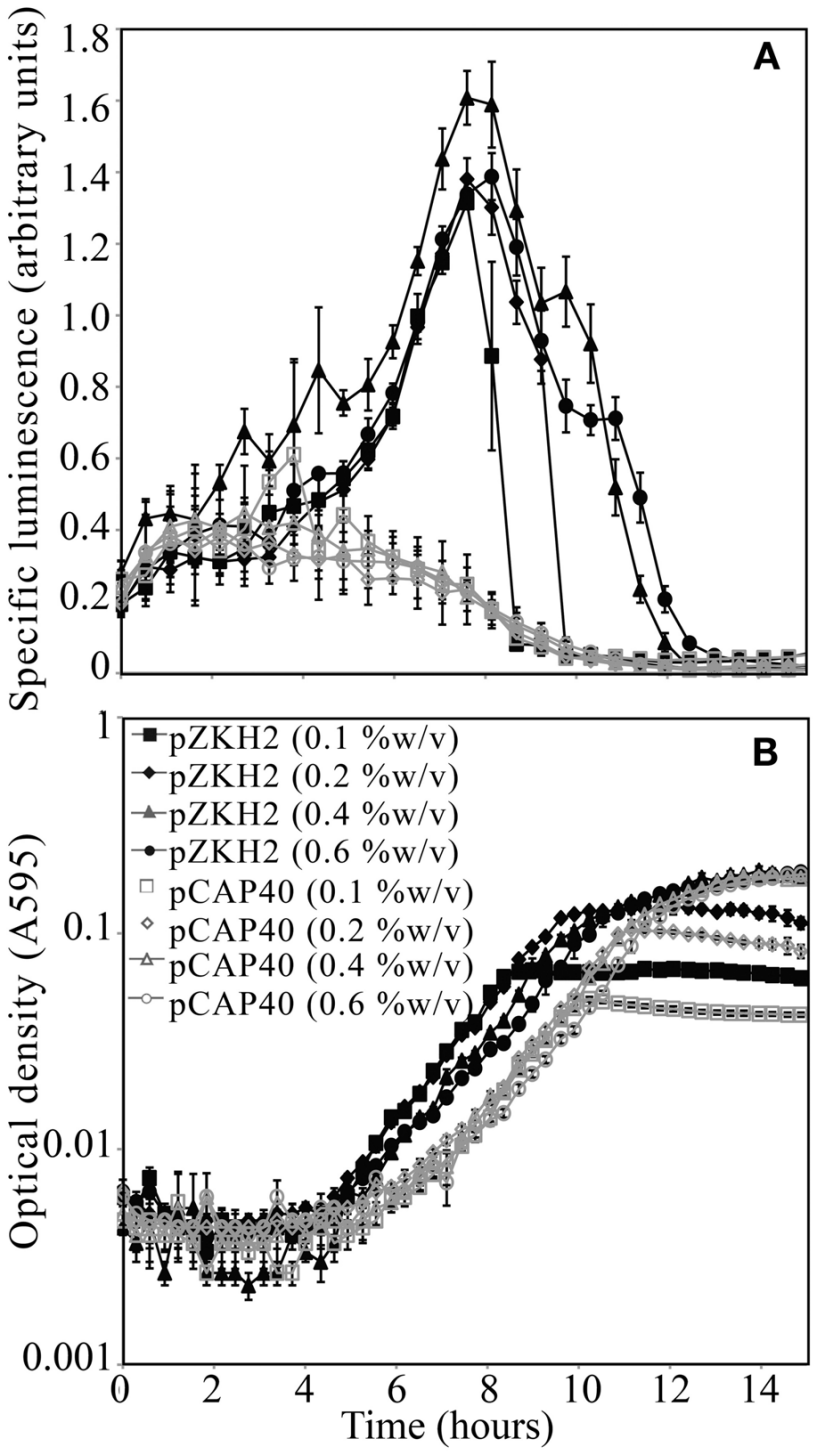
**(A)** Specific luminescence (×10^5^) from microbiosensor KT2440/pZKH2 (black lines, filled symbols) and promoter-less KT2440/pCAP40 (gray lines, open symbols) throughout exponential growth and into stationary phase tracked using **(B)** optical density (A595). Bacteria were inoculated into M9 medium with four different concentrations (%w/v) of citrate in 48-well plates. All values are mean ± S.E.

For the biosensor *P. putida* KT2440/pZKH2 (black lines), calculated specific luminescence (Figure 2A) peaked during mid-exponential growth (Figure 2B); specific luminescence decayed rapidly to zero in late log to stationary phase. Specific luminescence from the promoter-less construct *P. putida* KT2440/pCAP40 (gray lines) exhibited no mid-exponential growth peak (Figures 2A,B), but equaled specific luminescence from the biosensor both early in the growth cycle when cell numbers were very low, and in late log to stationary phase (when luminescence dropped to zero).

**Figure 3:**
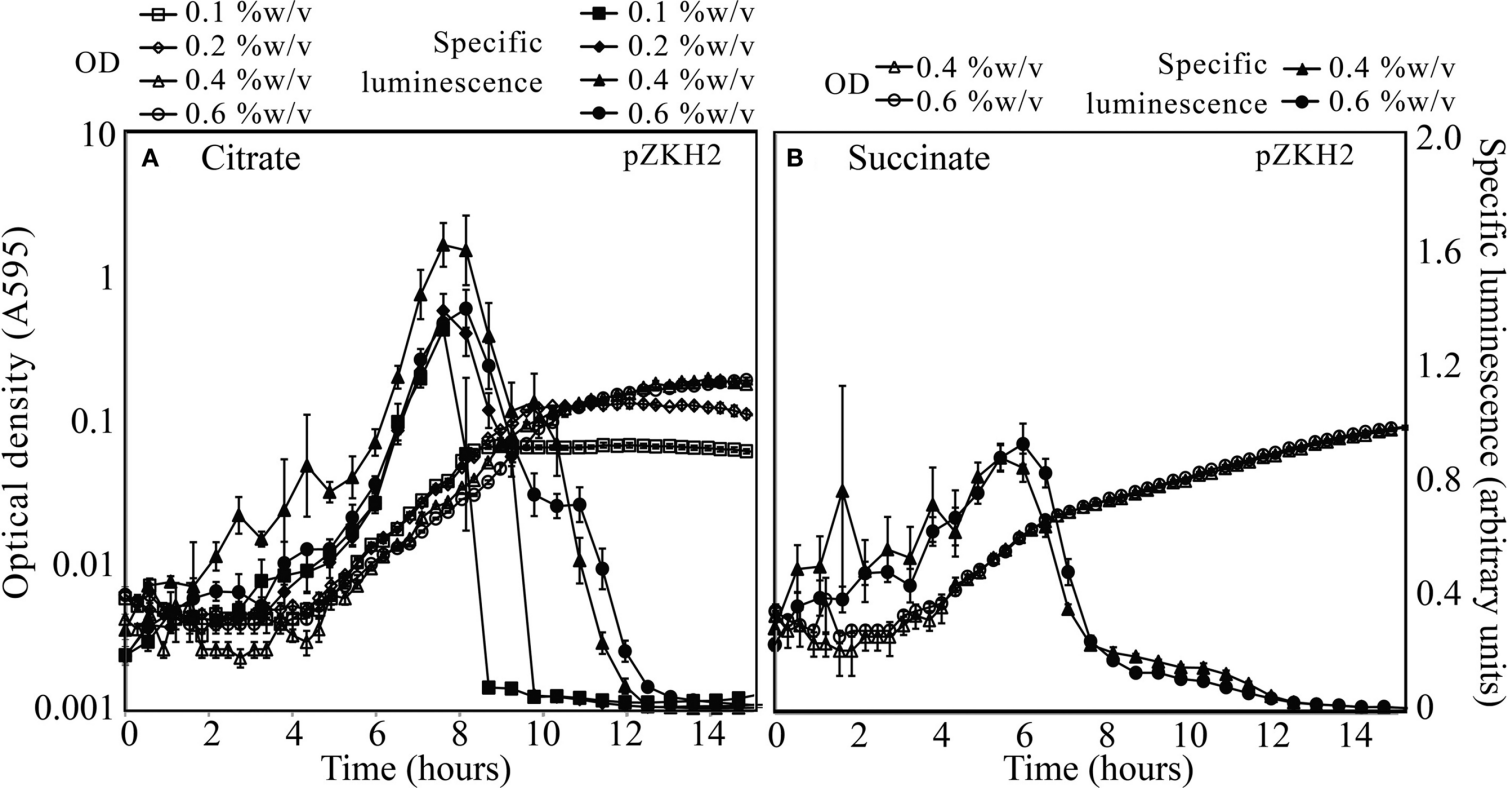
**Specific luminescence (×10^5^, filled symbols) and optical density (A595, open symbols) from microbiosensor KT2440/pZKH2 during growth in citrate (A) or succinate (B), in 48-well plates**. Bacteria were inoculated into M9 medium with citrate at one of four concentrations (%w/v) or succinate at one of two concentrations (%w/v). All values are mean ± S.E.

The mid-exponential peak in specific luminescence was observed for the biosensor *P. putida* KT2440/pZKH2 in both citrate and succinate, at all concentrations (Figures 3A,B). Peak luminescence persisted longer at higher concentrations.

#### Influence of carbon, M9, and oxygen availability on light production

**Figure 4:**
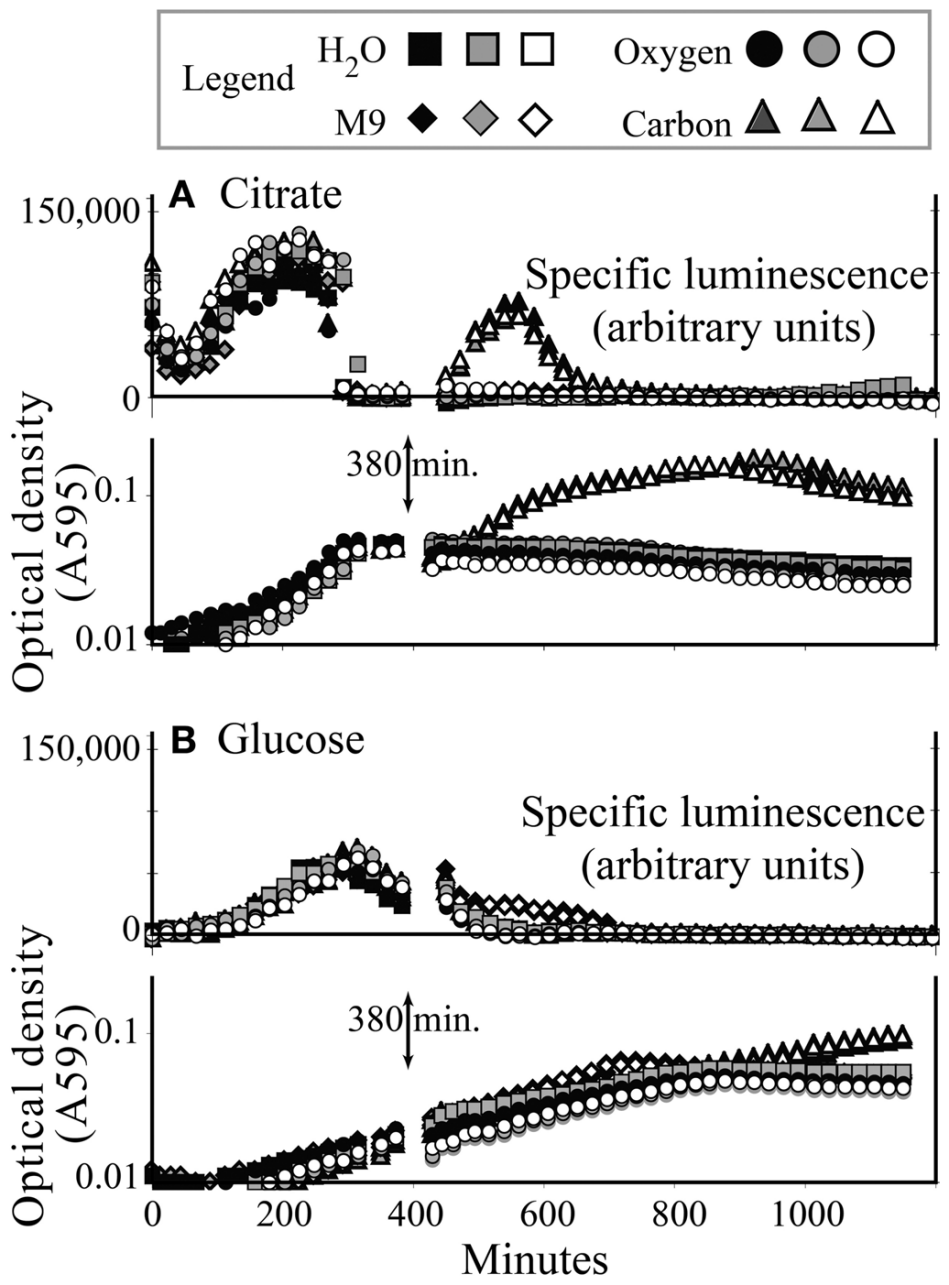
**Induction of specific luminescence from microbiosensor KT2440/pZKH2 during growth in citrate (A) or glucose (B), in 48-well plates**. At 380 min., triplicate wells were spiked with carbon, oxygen, M9 medium, or water. Growth and luminescence data are separated for clarity. Each replicate is shown separately.

Three hundred and eighty minutes into a typical growth experiment, KT2440/pZKH2 growing in M9+citrate had reached stationary phase, and specific luminescence had dropped to zero (Figure 4A). Specific luminescence increased again only when more citrate was added, which also spurred more growth (after a lag). Adding oxygen, mineral nutrients (M9) and water (a control) had no effect. In contrast, KT2440/pZKH2 growing in M9+glucose was still in late exponential phase at 380 min., and specific luminescence had only just begun to decline. Addition of more glucose did not increase luminescence and spurred only minimally more growth, hours later. Addition of O_2_, mineral nutrients, or water also had no effect (Figure 4B).

### Experiments on filter discs

#### Dynamic response to pulsed carbon availability

**Figure 5:**
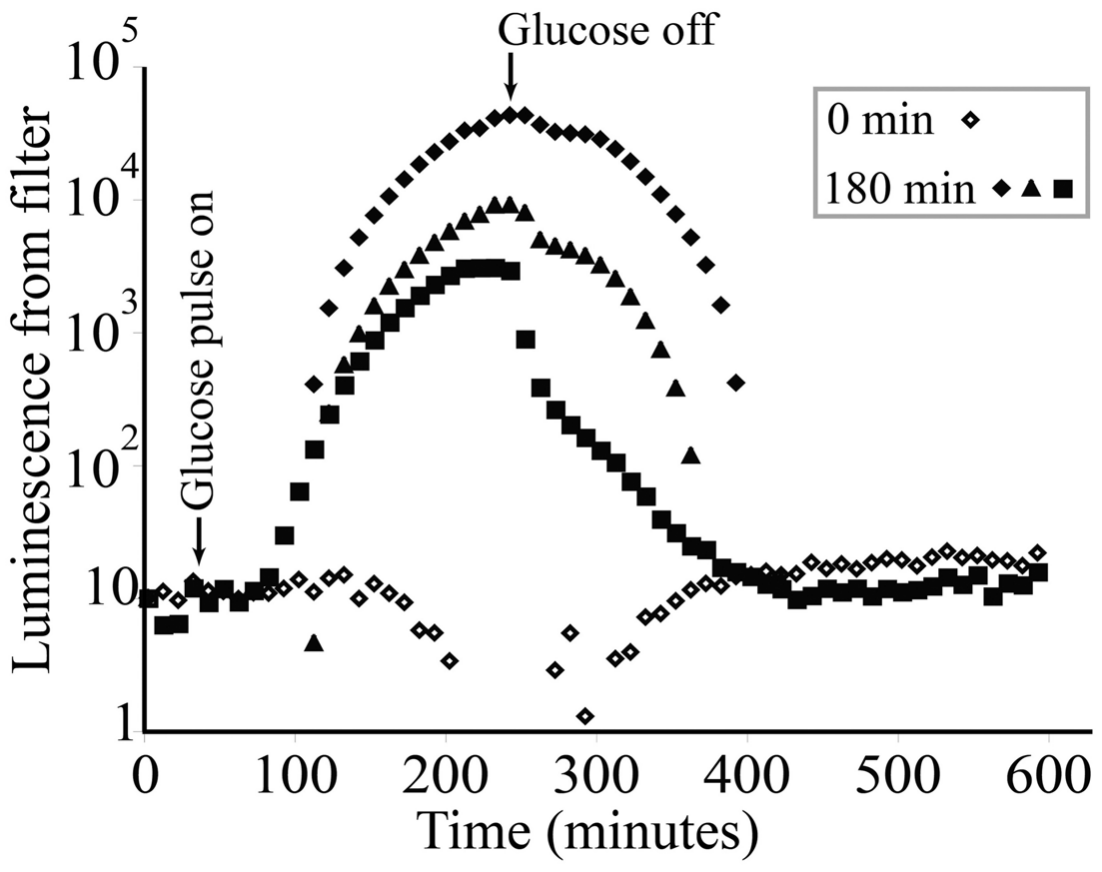
**Luminescence from populations of microbiosensor KT2440/pZKH2 established on 0.45 μm syringe filters and receiving M9 medium at a constant flow rate of 0.5 ml min^−1^**. Starting at approximately 40 min, 3 replicates were exposed to a 180-minute pulse of 0.1% (w/v) glucose in M9, then returned to M9 alone. Each glucose-treated replicate is shown separately to illustrate dynamics more clearly (0 min indicates no glucose added).

KT2440/pZKH2 biosensors deposited on filter discs and flushed continuously with M9 salts containing no glucose exhibited low light production through time (Figure 5, open diamonds). KT2440/pZKH2 biosensors exposed to 180 min of 0.1% glucose in M9 responded with strongly increasing specific luminescence, starting approximately 1 h after exposure to high glucose. Luminescence increased and had just reached a plateau when glucose flow stopped, then luminescence declined to baseline over approximately 2.5 h.

### Experiments in soil – no plants

#### Detection of biosensor luminescence in soil supported by distinct carbon substrates

Luminescence detected from soil is a function of specific luminescence and bacterial population size, both of which are likely affected by carbon substrates available to support growth. To test whether the camera systems we had available were sensitive enough to detect luminescence in soil, KT2440/pZKH2 was inoculated into soil in various combinations of cell densities, carbon types and concentrations, with each treatment loaded into a 96-well plate and imaged by a Retiga EX CCD camera.

**Figure 6:**
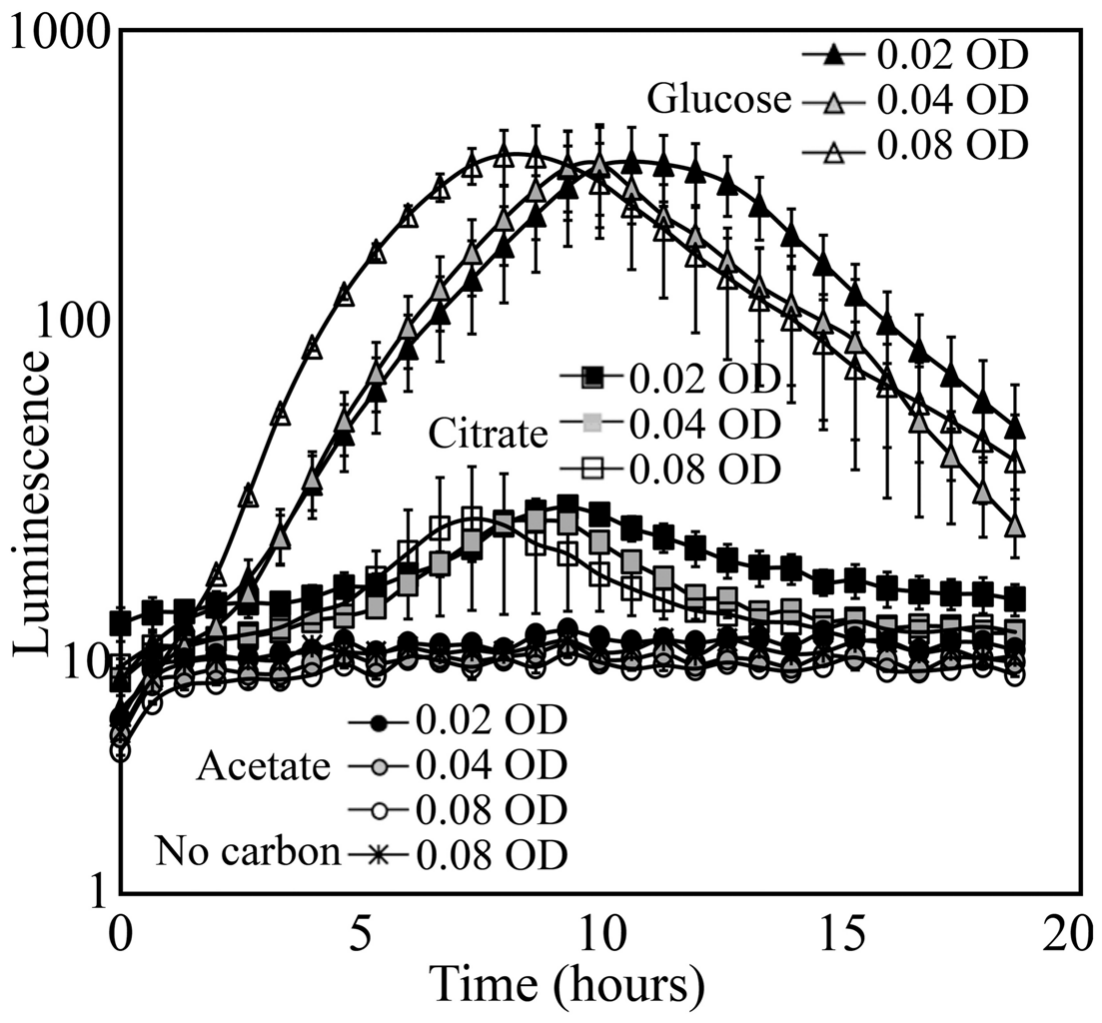
**Luminescence detected using a Retiga EX CCD camera from populations of the microbiosensor KT2440/pZKH2 established in soil in 96-well plates**. Bacteria were grown in M9 with 0.4% (w/v) citrate, glucose, or acetate as carbon source, spun down and re-suspended to three optical densities for each source, then inoculated into soil. All values are mean ± S.E.

KT2440/pZKH2 that was inoculated with M9+glucose medium into soil yielded strong luminescence that was easily detected by the camera; inoculation with citrate yielded detectable but much lower luminescence (Figure 6). Inoculation with M9+acetate resulted in no increase in luminescence, either because populations of luminescing bacteria were too small, or specific luminescence was low on acetate, or both. In the case of bacteria supplemented with media containing glucose or citrate, the timing of peak light production was related to the inoculation density. Inoculations at an OD of 0.08 yielded peak luminescence earliest, followed by the inoculations of 0.04 and 0.02 OD, as would be expected from a combined population-size effect tempered by decreasing light production as bacteria approach stationary phase.

#### Response of biosensor luminescence in soil to repeated pulses of carbon and mineral nutrients

**Figure 7:**
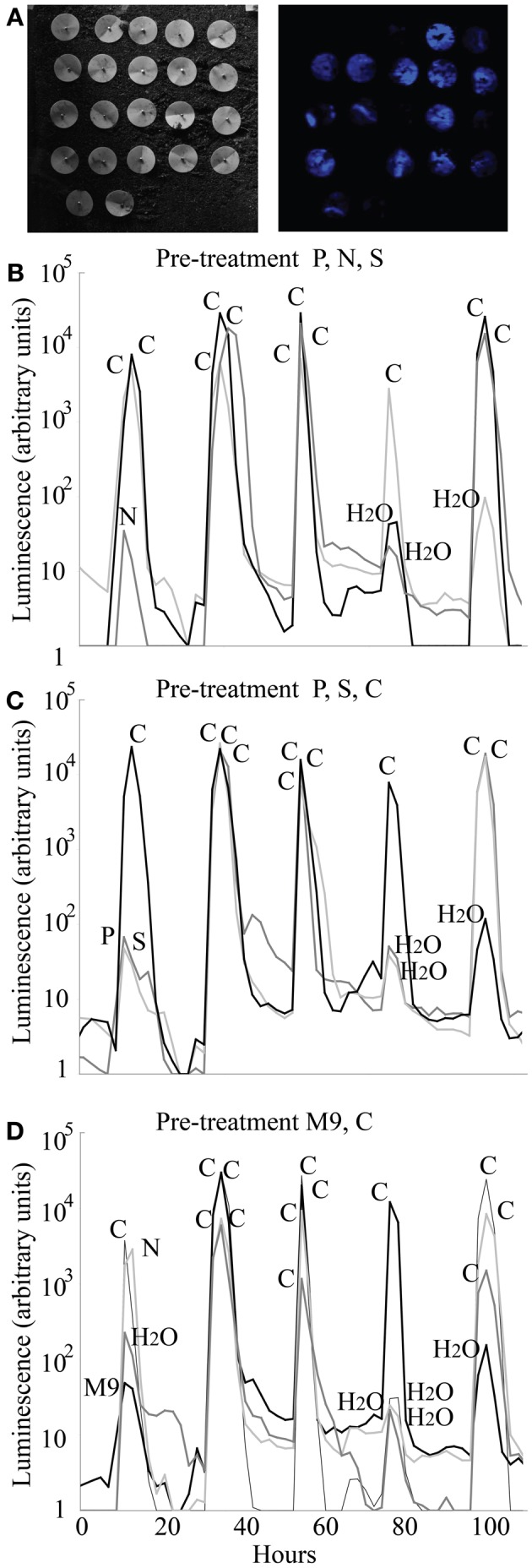
**Luminescence detected using a Versarray CCD camera from populations of microbiosensor KT2440/pZKH2 inoculated onto soil and provided with pulses of carbon, various mineral nutrients, and water, all delivered via filter discs pinned to the soil surface**. Panel **(A)** shows brightfield (left) and false-color luminescence (right) images of the filters. Panels **(B,C,D)** show luminescence from replicate filter discs within three of seven pre-treatments (see text), over five days, as pulses of carbon, nutrients, and water were applied.

We tested the ability of the Princeton Instruments cooled Versarray CCD camera to detect luminescence from biosensors in soil, and the repeatability of the KT2440/pZKH2 biosensor response in soil to pulses of carbon, nutrient, and water availability provided via filter discs, over multiple days. Only data from filter pre-treatment groups 5, 6, and 7 are shown in Figure 7; pre-treatment groups 1, 2, 3, and 4 yielded similar results. No matter the pre-treatment, and no matter the day, addition of glucose to any filter resulted in orders of magnitude increase in luminescence (Figure 7, all peaks labeled “**C**”). Small increases in luminescence were induced by water (e.g., see 80 and 100 h, panels 7B,C,D) and by mineral nutrients delivered in water (e.g., N at 10 h, panel 7B; P and S panel 7C at 10 h; M9 panel 7D at 10 h). Additions of these solutions may have mobilized small pockets of soil carbon that were previously unavailable to the biosensors.

One treatment, on one day, yielded an anomalous result—the addition of N on the first day after the pre-treatment of M9 + C (Figure 7D). It is possible that the bacteria were nitrogen limited before the addition of the N, but the discs that received M9 with an equivalent level of nitrogen did not ramp up light production significantly. The anomalous result is likely either a case of carbon being mobilized by the liquid addition of N from surrounding pockets not in contact with the bacteria, or the experimenter adding carbon in that spot by accident.

### Experiments in soil – soil microcosms with plants

KT2440/pZKH2 produced light on all type of plants that were inoculated including corn (*Z. mays* L.), tomato (*S. lycopersicum* L), pepper (*C. annuum* L., data not shown), sagebrush (*Artemisia tridentata* var. *vaseyana*, data not shown) and black poplar (*P. nigra* L.). The biosensor produced similarly high intensities of light when inoculated on corn, tomato and pepper (pepper data not shown). When inoculated on sagebrush (data not shown), whose overall growth rate is not as fast as the crop species used, KT2440/pZKH2 produced lesser but easily measurable amounts of light. KT2440/pZKH2 produced the smallest output of light on *P. nigra*. Selected results from corn, tomato, and poplar are described in detail below.

#### Corn

**Figure 8:**
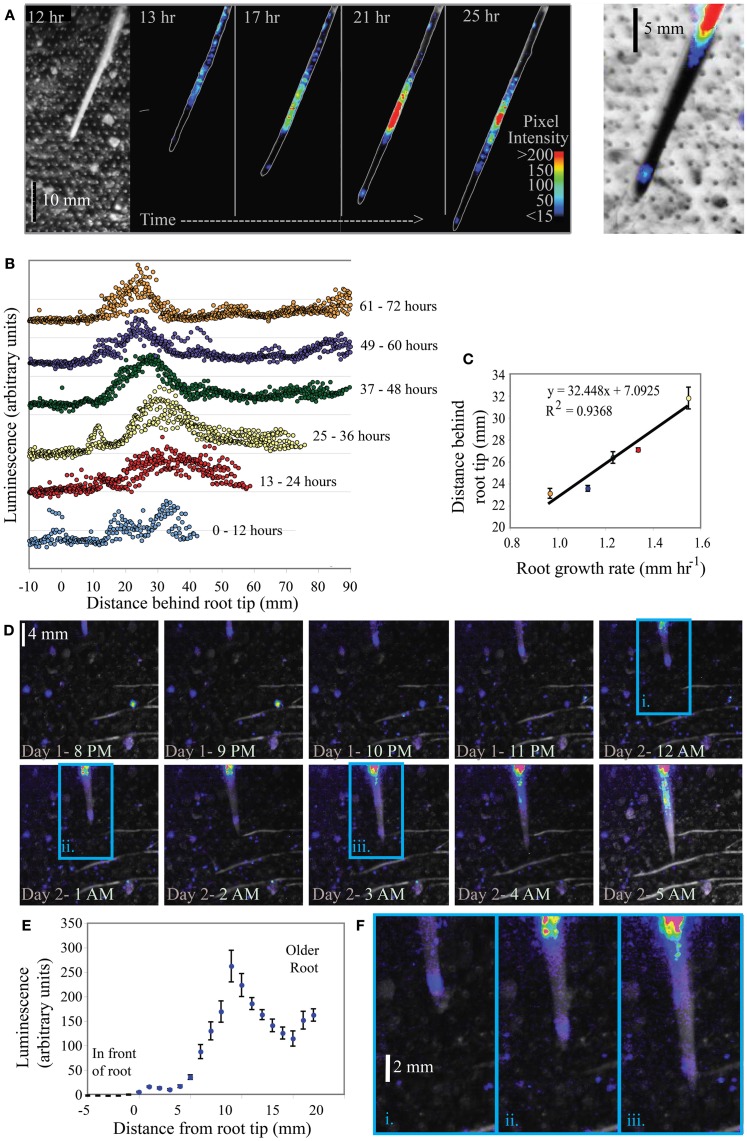
**Luminescence from microbiosensor KT2440/pZKH2 inoculated into microcosms containing 3-week old *Zea mays* (corn)**. Panel **(A)** shows a bright field (left) and a small subset of images from a time series of false-colored luminescence around a single growing corn root, along with a closeup (right) of false-color luminescence superimposed on an inverted brightfield image of the corn root tip. Panel **(B)** charts luminescence quantified hourly along the root, binned into six time frames. Panel **(C)** graphs the distance of maximum luminescence from the root tip against root growth rate. Panel **(D)** shows a portion of a similar image series from another corn plant, and panel **(E)** shows the distribution of luminescence along the root axis averaged over the 25 h experiment. Panel **(F)** includes zoomed portions of images from the panel **(D)** series (indicated by i, ii, and iii), showing false-color luminescence close behind the growing root tip.

KT2440/pZKH2 consistently emitted a high amount of light on growing corn roots. Results from monitoring two corn plants are shown in Figures 8A–F. Both image series demonstrate a clear pattern of light emission associated with the tips of corn roots as they grow through the soil. For plant 1 (Figures 8A–C), the major peak of light emission occurs 22–32 mm behind the growing root tip along the root axis, though another peak in luminescence also occurs ~1–4 mm behind the root tip. A bright-field image of the root is shown at left; the regular grid of dots is the grid of air holes in the bread bag covering. Luminescent regions are visible from the false color superimposed on the outline of the corn root in all four black panels in Figure 8A. Intensity of luminescence is indicated by color, with lower values blue, increasing through yellow and then red for highest luminescence. Figure 8B shows the distribution of luminescence along the root at 1 mm intervals, from 0 to 90 mm behind the tip, with hourly measurements binned into 12-h increments. These binned data are separated along the Y axis for clarity, with earlier times lower, progressing to later times in the experiment higher in the graph. As time progressed, soil drying slowed root growth and the peak of light emission shifted closer to the tip (Figure 8B). The distance between the peak of light emission and the root tip is linearly related to the growth rate of the root (Figure 8C). Similar results from a second corn plant are shown in Figures 8D–F.

#### Black poplar

**Figure 9:**
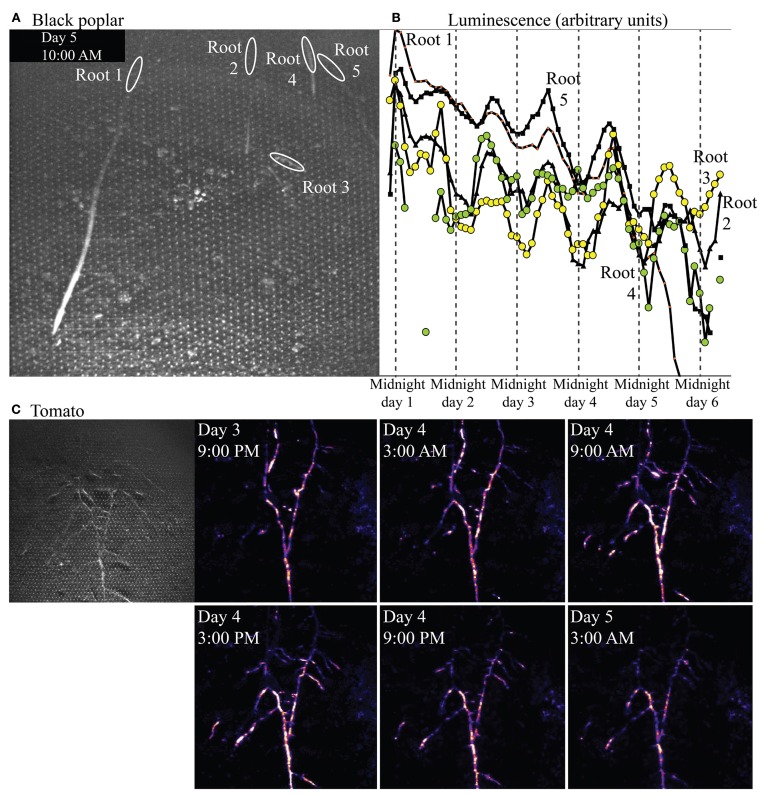
**Bright-field image showing roots of *Populus nigra* (black poplar) established in a soil microcosm, and the rhizosphere regions of interest associated with mature zones of five roots**. Panel **(B)** shows luminescence quantified from those regions over time. Panel **(C)** shows a brightfield (left) and time series of false-color luminescence images (left to right, top row then lower row) from a microcosm planted with *Solanum lycopersicum* (tomato).

Figure 9A shows a bright-field image of one microcosm planted with *P. nigra*, and the regions of interest (ROIs) defined in ImageJ from which luminescence was quantified over time, and graphed in Figure 9B. The ROIs illustrated are in root locations that are mature; tissue is no longer extending or expanding but the root is still young (not woody) and cortex is still intact. Though overall luminescence from biosensors around these mature regions decreased over the 6 days of the experiment, the biosensor luminescence associated with all five roots also exhibited a strong diel rhythm throughout the experiment.

#### Tomato

Figure 9C shows a bright-field image of an established, mature tomato root system (at left), and a subset of a series of luminescence images taken over 4 days. Luminescence images progress in time left to right, top row then bottom row, within Figure 9C, spaced at 6 h intervals. Biosensor luminescence was highly dynamic, progressing in acropetal waves along root axes during the course of the experiment. A video of luminescence around this tomato root system is included in online supplementary materials.

## Discussion

Interactions among roots and soil microorganisms occur within a complex, heterogeneous matrix of soil grains and organic matter. Organic compounds are released and root cap cells distributed into the soil dynamically as root systems become established. These compounds influence soil bacteria and fungi at spatial scales difficult to measure, with dynamics that cannot be captured with destructive soil sampling techniques. Experiments simplifying the root-microbial system by isolating plants and roots into hydroponic or other culture systems have been valuable for detecting diel patterns of root exudation, types of compounds released from roots into solution (and the effects of the presence of microbes on that release), and signaling compounds exchanged by roots and microbes. Microbial biosensors used in non-sterile soil complement these approaches by providing information on local microenvironmental resources and conditions experienced by the microbes themselves.

Microbiosensor design is flexible and can be tailored to particular questions via choice of host organism, promoter, and reporter genes (Gage et al., 2008). Here we used a host bacterium that is native to the rhizosphere environment, and a reporter system with a constitutive promoter (*nptII*) fused to the *luxCDABE* operon. The great strength of the system is that the light output that emerges reflects the growth and metabolic activity of a soil bacterium that is native to the rhizosphere. It might be expected that because the *nptII* promoter is constitutive, luminescence on a per-cell basis would be relatively constant during microbial growth. However, the results of liquid culture experiments (Figures 2–4) revealed a strong peak of light production at mid-exponential growth followed by the rapid decrease in light production as cells moved into stationary phase. Light production by these microbiosensors is therefore a signal that all conditions and resources in the biosensors' local environment are conducive to (and supporting) rapid growth.

The decrease in light production initiated as cells move from late exponential to stationary phase is likely connected with the high energy demand of bioluminescence. *P. putida* KT2440/pZKH2 contains the full *lux* operon *luxCDABE*. Light production requires the dual oxidation of a reduced flavin mononucleotide (FMNH_2_) and an aldehyde molecule (RCHO). *luxA* and *luxB* code for the alpha and beta-subunits of the luciferase enzyme responsible for carrying out that oxidation. *luxCDE* encode a multi-enzyme reductase complex responsible for the regeneration of aldehyde (RCHO) (Meighen, 1988), requiring one NADPH and ATP (Stryer, 1988). Overall, close to 20 ATP molecules are estimated to be required to produce one quantum of light (van der Meer et al., 2004), imposing a significant metabolic burden on bacteria.

Results from multiple experiments are consistent with the idea that the *lux* system is competing with other cellular activities for energy. Our working hypothesis for the growth stage dependent behavior of the biosensor is that as cells transition from late exponential growth toward stationary phase, competition within the cells for dwindling pools of energy intensifies and light production rapidly decreases (e.g., Figure 2). Figure 4A shows that when KT2440/pZKH2 growing in M9+citrate had reached stationary phase, specific luminescence increased again very rapidly when more citrate was added (suggesting *lux*-related machinery was still present and ready to act when energy became available), whereas growth resumed after a short lag. The contrasting behavior of biosensors spiked with glucose in liquid culture, Figure 4B, where no increased luminescence or growth were observed when glucose was added, resulted because the cells were still growing exponentially; they had not reached stationary phase. Biosensors already had sufficient resources to grow exponentially, they were already highly luminescent, and neither growth rate nor luminescence increased quickly with added glucose. However, repeated application of glucose to filter disks pinned to soil seeded with KT2440/pZKH2 resulted in consistent pulses of light production, over a number of days (Figure 7), consistent with the idea that carbon limits microbial growth in bulk soil (Cardon and Gage, 2006).

Studies on *E. coli* carrying a plasmid with a full complement of *lux* genes (*luxCDABE*) (Rattray et al., 1990) found a similar effect of growth stage influencing overall light production. However, when Rattray et al. (1990) exogenously supplied dodecyl aldehyde to *E. coli* strains carrying only *luxABE* on the plasmid, the strain was able to express light consistently across growth stage. The supply of aldehyde subverted the dependence of the luciferase reaction on fatty acids diverted away from the cells' normal lipid production.

The amount of luminescence from microbiosensors in soil recorded by the camera system is influenced by both the luminescence per cell (specific luminescence, influenced by promoter activity, *lux* machinery turnover, and energy pool sizes) and by the population size of the bionsensors per unit area. We imaged at relatively low magnification to capture behavior of the luminescent signal emanating from populations of bacteria, through time, across entire root systems or for millimeters behind growing root tips. Light production was informative; darkness was not. Where the camera detected light, conditions and resources must have been conducive for growth and energy production, and populations of microbiosensors must have built to sufficient size to produce detectable signal. Darkness, in contrast, could result from e.g., insufficient resources for light production, conditions not conducive to growth of sufficient numbers of microbiosensors, and death of sensors (grazing protozoa were present in these non-sterile soils).

Within these constraints for interpretation, our results from soil microcosm experiments with plants are consistent with biosensors reporting conditions in which rapid growth can occur. Not surprisingly, luminescence was most often seen clearly associated with live roots for all plants tested—the rhizosphere is a hotspot for microbial activity in ecosystems, where carbon fixed by plants and released or lost by roots spurs microbial growth and activity. Plant species-specific patterns, however, were also observed.

Rhizodeposition by corn roots is known from the literature to be very high (McCully, 1999), and in our experiments was substantial enough to support bright luminescence even within millimeters of the root tip. Again, to detect this luminescence using the camera, not only did biosensor cells need to luminesce, but biosensor population sizes had to be large enough that the camera could detect light from them. The clear linear dependence of the location of maximum luminescence (along the root axis) on root growth rate is consistent with it taking time for the population of bionsensors to build up sufficiently to produce maximum detectable luminescence. The slower the root grows, the closer to the tip are locations that have been exposed to high rhizodeposition for long enough for a microbial population to grow larger. All plant roots grow by adding new cells at the tip, building root tissue at the tip through soil (Raven and Edwards, 2001). A root growth rate of 1 mm h^−1^ (as in corn Figure 8C) is a measure of the position of the root tip in soil over time, but does not represent a pushing of the entire long root axis through soil at that rate. As cells are built on the tip of the root, the cells behind the tip generally remain in place in soil (though they do expand before maturing). Using the growth rate and maximum luminescence data in Figure 8C, we can estimate that the position at which maximum luminescence occurs along the root is approximately 22–23 h old (average 22.5 ± 0.6 h SE, for the five points shown in Figure 8C). Interestingly, using the high magnification images in Figures 8A,F, it is clear there is another, but less bright, “hotspot” of luminescence just 1–4 mm behind the root tip. Again using root growth rate data, we can calculate that this location is just a few hours old, yet resources there are high enough that luminescence is detectable. This time frame is consistent with the data from labile carbon pulse experiments in Figure 5, where an increase in glucose available to filter-immobilized bacteria resulted in increased luminescence detectable by a camera after approximately 1 h.

Results from black poplar, however, provide an informative contrast. Luminescence was never detected by the camera at or near the growing tips of poplar roots, and luminescence overall was very low around all roots. We suspect this may be because populations of bionsensors did not build up to sufficient sizes, rapidly enough, for detection at the tips; we have noted in other experiments that *Sinorhizobium*-based microbiosensors do not proliferate easily around roots of poplars, for as yet unknown reasons. However, low luminescence was detected around mature regions of the roots, and quantification of that luminescence over time revealed a very strong diel cycle over several days (Figure 9B), with peak luminescence near noon, and lowest luminescence near midnight. Strong diel patterns in allocation of carbon from shoots to roots are known in the forestry literature for species within the genus *Populus* (e.g., Dickson, 1991). Starch builds up in leaves during the day, then is mobilized and carbon shipped belowground as sucrose at night. We do not yet know whether such a strong pattern of daytime starch buildup and nighttime starch mobilization exists in black poplar, but the diel pattern in luminescence (suggesting rhythmic diel carbon availability in the rhizosphere) that we observed would be nearly half a day out of phase with such a diel carbon allocation pattern within the plant.

In contrast to this coordinated, rhythmic but very low biosensor luminescence along multiple poplar roots, luminescence from bionsensors associated with tomato roots was bright and did not follow a diel rhythm. The luminescence pattern observed day 5 at 3 AM, for example, is different from the pattern observed day 4 at 3AM (Figure 9C). Biosensor luminescence progressed in waves along root axes acropetally (toward root tips) during the course of the experiment. The supplementary video shows an initial surge of light from all areas of the microcosm as microbiosensors use available resources, eventually settling into a pattern of dynamic luminescence restricted largely to the rhizosphere.

## Conclusion

It has long been known that different plant roots release different kinds of carbon compounds, that shoot-root allocation patterns vary widely, and that plant roots grow at different rates depending on environmental conditions. Biosensor *Pseudomonas putida* KT2440/pZKH2 adds an integrated microbial report on whether rhizosphere conditions and resources in the Pseudomonads' local soil microenvironment are supporting rapid growth. From the perspective of the rhizosphere being a key commodities exchange in ecosystems, this integrated view is essential—many resources may support microbial growth, and a range of conditions exist around plant roots. KT2440/pZKH2 luminescence indicates that energy is available for bacterial growth around roots beyond the moment that the root tip grows past a particular site. KT2440/pZKH2 luminescence also reveals that not all root tips are equal. Though a poplar root tip may be similar in size to a root tip of corn or tomato, it drives very different microbial response; root biomass is not necessarily a strong predictor of the capacity of a root system to spur microbial growth and activities. KT2440/pZKH2 also reports that availability of carbon is variable in space and time around plant roots, sometimes in a coordinated or predictable spatial or temporal pattern based on plant allocation or vasculature. But no matter the ecosystem function of interest, sufficient microbial biomass hosting the genetic capacity for that function must build up before the microbes' effects can be exerted.

### Conflict of interest statement

The authors declare that the research was conducted in the absence of any commercial or financial relationships that could be construed as a potential conflict of interest.
